# The Effect of NeuroMuscular Electrical Stimulation on Quadriceps Strength and Knee Function in Professional Soccer Players: Return to Sport after ACL Reconstruction

**DOI:** 10.1155/2013/802534

**Published:** 2013-12-05

**Authors:** J. Taradaj, T. Halski, M. Kucharzewski, K. Walewicz, A. Smykla, M. Ozon, L. Slupska, R. Dymarek, K. Ptaszkowski, J. Rajfur, M. Pasternok

**Affiliations:** ^1^Department of Physiotherapy Basics, Academy of Physical Education in Katowice, Mikolowska Street 72, 40-065 Katowice, Poland; ^2^Department of Medical Biophysics, Medical University of Silesia in Katowice, Medykow Street 18, 40-752 Katowice, Poland; ^3^Department of Physiotherapy, Public Higher Professional Medical School in Opole, Katowicka Street 68, 40-060 Opole, Poland; ^4^Department of Descriptive and Topographic Anatomy, Medical University of Silesia in Zabrze, Jordana Street 19, 41-808 Zabrze, Poland; ^5^Department of Physiotherapy, University of Medicine in Wroclaw, Grunwaldzka Street 2, 50-355 Wrocław, Poland; ^6^Department of Nervous System Diseases, University of Medicine in Wroclaw, Bartla Street 5, 51-618 Wrocław, Poland; ^7^Department of Gynecology and Obstetrics, University of Medicine in Wroclaw, Bartla Street 5, 51-618 Wrocław, Poland

## Abstract

The aim of this study was to assess the clinical efficacy and safety of NMES program applied in male soccer players (after ACL reconstruction) on the quadriceps muscle. The 80 participants (NMES = 40, control = 40) received an exercise program, including three sessions weekly. The individuals in NMES group additionally received neuromuscular electrical stimulation procedures on both right and left quadriceps (biphasic symmetric rectangular pulses, frequency of impulses: 2500 Hz, and train of pulses frequency: 50 Hz) three times daily (3 hours of break between treatments), 3 days a week, for one month. The tensometry, muscle circumference, and goniometry pendulum test (follow-up after 1 and 3 months) were applied. The results of this study show that NMES (in presented parameters in experiment) is useful for strengthening the quadriceps muscle in soccer athletes. There is an evidence of the benefit of the NMES in restoring quadriceps muscle mass and strength of soccer players. In our study the neuromuscular electrical stimulation appeared to be safe for biomechanics of knee joint. The pathological changes in knee function were not observed. This trial is registered with Australian and New Zealand Clinical Trials Registry ACTRN12613001168741.

## 1. Introduction

One key goal of successful anterior cruciate ligament (ACL) reconstruction is return to sports. Athletes who sustain ACL tears require successful reconstruction in order to continue participating in cutting and pivoting sports. The two most commonly reported treatment options for ACL injuries in sport is surgical operation and physical therapy management [1].

Quadriceps muscle weakness after anterior cruciate ligament (ACL) reconstruction is a serious problem, but the mechanisms underlying these chronic strength deficits are not clear. For example, Krishnan and Williams [2] examined quadriceps strength in people 2–15 years after ACL reconstruction and tested the hypothesis that chronic quadriceps weakness is related to levels of voluntary quadriceps muscle activation, antagonistic hamstrings moment, and peripheral changes in muscle. Knee extensor strength and activation were evaluated in 15 ACL reconstructed and 15 matched uninjured control subjects using an interpolated triplet technique. Electrically evoked contractile properties were used to evaluate peripheral adaptations in the quadriceps muscle. Antagonistic hamstrings moments were predicted using a practical mathematical model. Knee extensor strength and evoked torque at rest were significantly lower in the reconstructed legs (*P* < 0.05). Voluntary activation and antagonistic hamstrings activity were similar across legs and between groups (*P* > 0.05). Regression analyzes indicated that side-to-side differences in evoked torque at rest explained 71% of the knee extensor strength differences by side (*P* < 0.001).

Adams et al. [3] noticed that quadriceps strength deficit is significant after ACL injury, ranging from 15% to 40% (followup was obtained 502 patients at a mean of 14.1 years postoperatively). Regression analysis showed that the most statistically significant factor related to lower subjective scores was lack of normal knee extension and loss of normal flexion.

Neuromuscular electrical stimulation (NMES) is the application of electrical current to elicit a muscle contraction and seems to be helpful for strengthening the muscles. Use of NMES for orthopedic and neurologic rehabilitation has grown significantly in recent years and seems to be documented [1, 4–7].

The neurophysiologic principles on which the treatment is based have been studied in animal and human subjects. A nerve action potential may be elicited by a “command” originating in the motor cortex of the brain or by an electrically induced stimulus at the periphery. In either case, the mechanism of action potential propagation and release of synaptic transmitter substance is the same. A fundamental difference between the two mechanisms and the resultant muscle contraction exists in the recruitment order of individual motor units [7].

During a voluntary muscle contraction, smaller motor units composed primarily of type I (slow twitch fibers), fatigue-resistant fibers tend to be recruited first. Different motor units are recruited asynchronously. As some are relaxing, others are contracting and constant tension of the muscle is maintained. During electrical stimulation such as NMES (especially for 50–70 Hz frequencies), composed primarily of type II (fast twitch fibers), readily fatigable fibers are recruited first because their (larger diameter) motor nerves have low thresholds to electrical excitation [8, 9]. Motor units of similar thresholds lying superficially beneath the stimulating electrodes will be recruited simultaneously. As they begin to fatigue, tension in the muscle will begin to decrease unless the intensity of the stimulus is increased, recruiting additional motor units with higher thresholds or with similar thresholds, but more remote locations [9]. Excessive fatigue can be minimized during electrically induced muscle contraction by limiting the frequency at which the stimulus is applied and the duration of the contraction. Adequate rest periods between contractions will increase the likelihood that subsequent muscle contractions will be sufficiently strong. In the literature there are lots of theories (historical and modern) on mechanism of generating the action potential and muscle nerve conducting: electronic propagation, Hodgkin-Huxley theory, patch claim technique, or theory of channel activation [9, 10].

The goal of improving human performance in sport and exercise has been an extremely interesting topic for coaches, athletic trainers, physical therapists, exercise physiologists and athletes alike. Unfortunately, the utility of the NMES for sport application still remains controversial. For example, while many research studies find muscle strength and recovery after physical effort significantly improved by NMES [3, 4, 11–14], a few do not [8, 15, 16]. It is also unclear whether the NMES is completely safe. Some authors maintain that NMES could induce muscle damage or influence on function and biomechanics of the near articulations of the stimulated muscles [17].

Bax et al. [18] presented a systematic review and metaanalysis of randomized controlled trials to determine whether NMES is an effective modality for strength augmentation of the quadriceps femoris. A full content search for randomized controlled trials was performed in Medline, Embase, Cinahl, the Cochrane Controlled Trials Register,F and the Physical Therapy Evidence Database. Only maximum volitional isometric or isokinetic muscle torque in Nm was used as main outcome measure and final conclusions are unclear. Authors stated that further research should be directed toward identifying the clinical impact at activity and participation levels and the optimal stimulation parameters of this modality. Well-prepared and documented, prospective, controlled studies are needed.

The objective of this study was to assess the efficacy and safety of NMES program applied in soccer players (after ACL reconstruction) on the quadriceps muscle. The primary study endpoint was a comparison of the change in muscle strength between the stimulated and control groups. The secondary end point was the analysis between groups of the change of quadriceps muscle circumference and other measured parameters as predictors of safety (whether there are any pathological changes in knee function) after NMES therapy.

## 2. Materials and Methods

All participants provided informed consent to this project that was approved by the Local Institutional Review Board of the Medical University of Silesia in Katowice, Poland. Trial is registered in Australian and New Zealand Clinical Trials Registry (ACTRN12613001168741).

### 2.1. Participants and Randomization

The 80 professional male soccer players (The Soccer Academy—ten soccer clubs from 2nd and 3rd Polish League) participated in experiments to measure the clinical effectiveness and safety of electrical stimulation. Participating subjects met the following inclusion criteria.

(1) They underwent arthroscopy surgery as follows: initially, an anteromedial incision was made on the proximal tibia and the gracilis and semitendinosus tendons were detached from their insertions on tibia. Subsequently, the tendons were removed to fashion the graft for ACL reconstruction. A tibial canal was established; through this canal, the femoral canal was created under arthroscopy guide. Finally, the graft was passed through the canals as a single bundle.

(2) They spent 6 months after operation.

(3) They received the same rehabilitation program before NMES procedures: week 1 (CPM Machine—start at 0–30 degrees and increase with not less than 10 degrees per day, PROM Wall Slides Seated Active Assistive Knee Flexion Prone Dangle Passive resting extension with heel prop Patellar Mobilizations, SLR x3 Flexion, Adduction, Abduction, Hamstring/Calf Stretches, Ankle Pumps, Gait Training, Home Exercise Program-2 or 3 times per day). Week 2–4 (Scar Mobilization/Massage, Proprioceptive Neuromuscular Facilitation, Progressive Resistive Exercises, Manual/Machine resisted leg press, Balance/Proprioception, Isometric Knee extension 90–60°, Stationary Bike, Minisquats progress up to 90°, Step ups, Retro Treadmill/Stairmaster, Hip abduction/external rotation) and week 4–16 (Functional Strengthening, Proprioceptive Neuromuscular Facilitation, Progressive Resistive Exercises, Manual/Machine resisted leg press, Balance/Proprioception, Squats to 90 degrees, Single leg squats, Step ups, Retro Treadmill/Stairmaster, and Review Home Exercise Program 2 times per day). Week 16–24 (Continue strength, endurance, proprioception progression, Begin double-leg hopping, jogging, and agility drills).

(4) They provided written informed consent to participate in the study.

Subjects with the following conditions were not allowed to participate or were excluded from the study:

1. women,
2. soccer practice for less than 3 years (share of the league for at least three years),
3. bone fracture in the last 2 years,
4. chronic sprain ankle,
5. a history of Achilles tendon injury.

Participants were randomly allocated to the groups. Computer-generated random numbers were sealed in sequentially numbered envelopes, and the group allocation was independent of the time and person delivering the treatment. The physician (research coordinator) who allocated the subjects to groups had 80 envelopes, each containing a piece of paper marked with either group A (NMES) or B (control). The physician would select and open an envelope in the presence of a physiotherapist to see the symbol and would then direct the patient to the corresponding group. Another physiotherapist collected the data and coded them into an Excel database. The “blinded” results were transferred to a Statistica version 10.0 database by a technician. The research coordinator had no contact with and could not identify the soccer players.

### 2.2. Procedures

All participants received an exercise program, including three sessions (Mondays, Wednesdays, and Fridays) weekly, for one month:

- double-leg hopping, jogging, agility drills, and free running,
- single-leg plyometrics, cutting/pivoting drills with stutter step pattern, high intensity aerobic/anaerobic sport specific training, and advanced lower extremity strengthening,
- triple hop for distance, single hop for distance, lateral hop 12′′ × 12′′ squares separated by 12′′ of hops (in box), and unilateral vertical jump.

Weight training exercise in the gym was prohibited.

**Figure 1:**
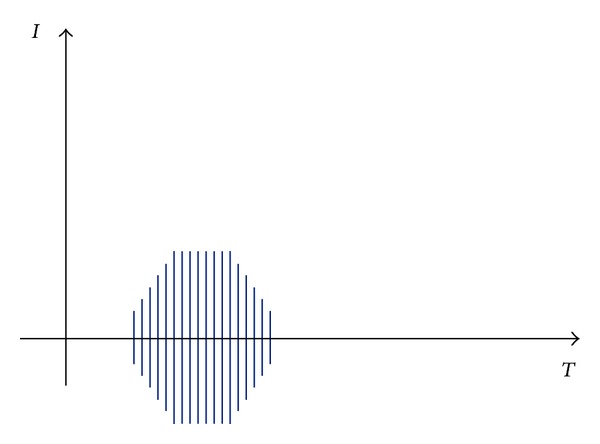
NMES impulse shape and characteristics. Legends: Base frequency: 2500 Hz, burst frequency: 50 Hz, on: 10 ms, off: 10 ms, increasing/decreasing ramp shape.

The individuals in group A additionally received neuromuscular electrical stimulation procedures on both right and left quadriceps (two electrodes on the muscle attachment sites). A portable electrical stimulator (Ionoson, Physiomed, Germany) delivered biphasic symmetric rectangular pulses (frequency of impulses 2500 Hz, train of pulses frequency 50 Hz). The stimulus output is interrupted every 10 ms to create “bursts” of stimulation every second. The 10 ms off period was not detectable by the subject. A total of 10 maximal contractions sustained for 10 seconds each with a 50 second off time defined a treatment session (according to methodology of stimulation prepared by Yakov Kots in year 1989 [10]—called in literature “Russian stimulation” and recognized as one of the types of the NMES—Figure 1). The intensity was between 55 and 67 mA (mean of 58.89 mA). Stimulation was performed with a current which produced strong, visible motion effects. Electrodes were made of conductive carbon rubber (8 × 6 cm). Before application of electrodes the skin was cleaned by use of alcohol. The total time of single procedure was 30 minutes. Quadriceps was stimulated at 60° of knee flexion. The procedures were repeated three times daily (3 hours of break between treatments), 3 days a week (Tuesdays, Thursdays, and Saturdays), for one month.

### 2.3. Measures

**Figure 2:**
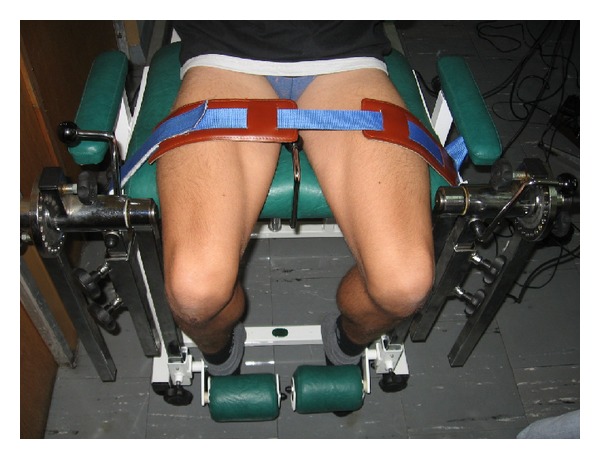
Tensometry.

Measurements of muscle strength were performed by a tensometer (Accuro Sumer, Poland). The tensometer was composed of resistance lever, roller, and electronic momentum meter (Figure 2). The spectrum of measurement was between 0 and 500 Newton meters (Nm). The magnitudes of force moment were converted into Newtons (N). The tested legs were at 60° of knee flexion (we observed maximal strength of muscle when individuals were prepared to the measurements).

Change of muscle circumference was measured by a “tailor tape” 10 cm above the patella on the quadriceps. The measurements were performed before and after experiment (the measurements were in a supine position before the first and after the last NMES procedure).

**Figure 3:**
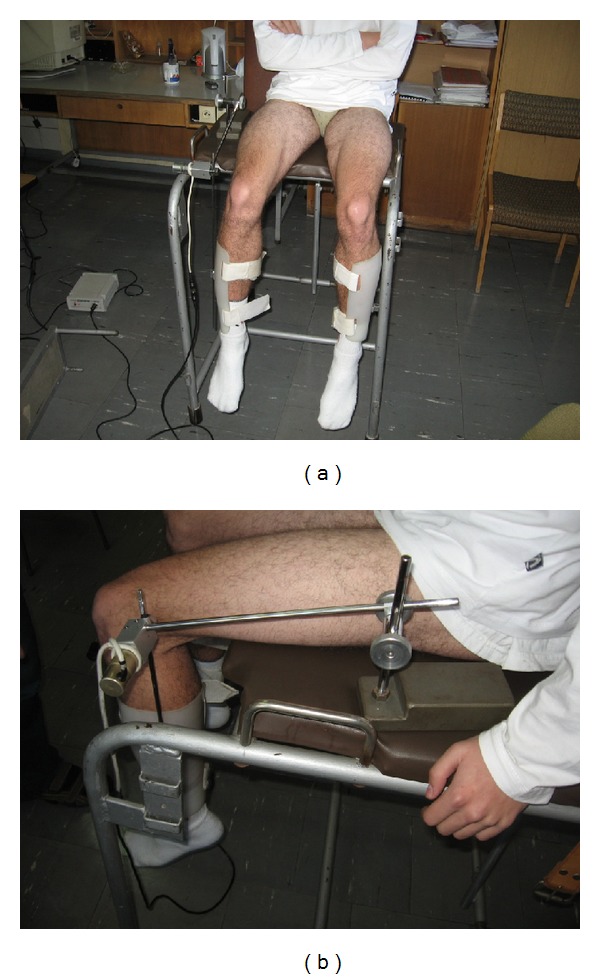
(a) and (b) are Goniometry pendulum test.

The goniometry pendulum test was composed of a peripheral device and a personal computer with software. The peripheral device consisted of a goniometry compass and an interface system-transducer cooperated with the computer. The goniometry compass was composed of two thin metal arms (Figures 3(a) and 3(b)). One of the arms (immobile) was fixed in a special outlet that allowed changing the angle and length. The mobile arm was connected to the athlete's leg. The motion of the compass was measured by a minioptoelectronic transducer. The position of the transducer was measured (with a resolution of 12 bytes (B) on one revolution) and coded in a range from 0° to 360° (exact to 0.088°). The signal was transmitted to the interface. The software allowed readings of 100 positions of the compass per second.

Using the pendulum test, the spectrum (range) and ease of motion in knee (whether the movement was smooth, physiological, and free in examined axis) were measured and determined by the following parameters:

 
*n*: number of oscillations,
 
*t*: whole time of oscillations,
 
*T*: period of oscillations (*T* = *t*/*n*),
 
*λ*: logarithm decrement of suppression (*λ* = ln⁡*X*_2_/*X*_4_, where *X*_2_ is a second amplitude during oscillations and *X*_4_ is a fourth amplitude during oscillations,
 
*β*: suppression index (*β* = *λ*/*T*).

**Figure 4:**
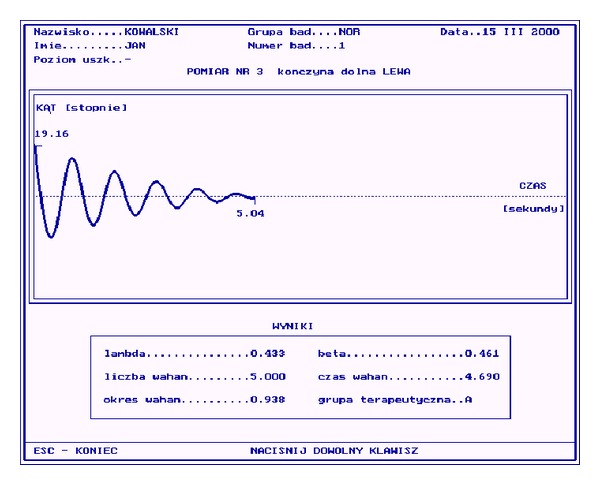
Exampled data from pendulum test (sinusoidal wave of pendulum movement). Legends: (lambda) *λ*: logarithm decrement of suppression. (beta) *β*: suppression index. (Okres wahan) *T*: period of oscillations. (Liczba wahan) *n*: number of oscillations. (Czas wahan) *t*: whole time of oscillations.

The number of oscillations (*n*) and whole time of oscillations (*t*) described the ease of knee motion. The period of oscillations (*T*) and suppression parameters (*λ*  and  *β*) assessed the spectrum of knee motion (Figure 4). The goniometry pendulum test was performed for analysis of NMES safety (to assess whether an increase in muscle mass and strength will change in the mechanics of the knee joint).

### 2.4. Follow-Up

The goniometry pendulum test was repeated 1 and 3 months after the study (79 athletes were measured in followup and only one participant from group B was excluded because of femoral neck fracture). The following procedure was performed for analysis of electrical stimulation safety in long term results.

### 2.5. Statistical Analysis

The chi-squared independence test (greatest reliability level) and parametric *t*-test were used for analysis of indicators, which characterized individuals in both comparative groups. The normal distribution was checked by Shapiro-Wilk (for the null hypothesis that a sample came from a normally distributed population) and the chi-squared test (when the null hypothesis was true, also consider that a chi-squared test is a test in which this is asymptotically true, meaning that the sampling distribution-if the null hypothesis is true can be made to approximate a chi-squared distribution as closely as desired by making the sample size large enough). Recalling that the null hypothesis is that the population is normally distributed, if the *P*-value is less than the chosen alpha level, then the null hypothesis is rejected (i.e., one concludes that the data are not from a normally distributed population).

If the *P*-value is greater than the chosen alpha level, then one does not reject the null hypothesis that the data came from a normally distributed population (according to statistical estimation the population over 30–35 is needed for further analysis of normal distribution, so we had to include about 80 participants in two groups in this study and use the parametric tests). Analyzed distributions appeared normal and we received the Gauss decay in all comparisons.

The parameters before and after study were compared in groups by parametric *t*-test (for dependent variables). Differences in parameters between groups were evaluated with *t*-test (for the independent variables); for comparisons of experimental and follow-up results we used analysis of variance ANOVA to analyze the differences between group means and their associated procedures (such as “variation” among and between groups). In ANOVA setting, the observed variance in a particular variable is partitioned into components attributable to different sources of variation.

Two-sided *P* values of less than 0.05 were considered to be statistically significant.

## 3. Results

The groups studied were homogeneous in terms of all participant characteristics (Table 1) and initial muscle power and knee biomechanical parameters. After experiment the strength of quadriceps on the operated side in group A (NMES) increased from 645.9 to 893.4 N (28.7%, *P* = 0.001) and in group B (control) it increased from 648.6 to only 669.8 N (4.6%, *P* = 0.04). The comparison after 1-month therapy of relative changes of the muscle strength between both groups showed a significant difference in favor of stimulation (28.7% versus 4.6%, *P* = 0.002, and 95% confidence interval). The strength of quadriceps on nonoperated side in group A increased from 840.1 to 1089.8 N (30.1%, *P* = 0.003) and in group B increased from 840.4 to only 885.2 N (4.6%, *P* = 0.04). The comparison after 1-month therapy of relative changes of the muscle strength between both groups showed a significant difference in favor of stimulation (30.1% versus 4.6%, *P* = 0.002, and 95% confidence interval).

In NMES group on the operated side quadriceps circumference increased from 56.5 to 57.9 cm (1.4%, *P* = 0.03) and in group B it increased from 56.2 to 57.1 cm (0.6%, *P* = 0.05). The comparison after a month of treatment of relative changes of the muscle circumference between both groups showed a significant difference in favor of stimulation (1.4% versus 0.6%, *P* = 0.04, and 95% confidence interval). On the non-operated side quadriceps circumference increased from 58.1 to 59.3 cm (1.1%, *P* = 0.03) and in group B increased from 57.7 to 58.2 cm (0.4%, *P* = 0.04). The comparison after a month treatment of relative changes of the muscle circumference between both groups showed a significant difference in favor of NMES (1.1% versus 0.4%, *P* = 0.04, and 95% confidence interval).

The comparison of all parameters measured using goniometry pendulum test in group A and B did not indicate any significant difference and also in the followup (after 1 and 3 months after the end of study)—Tables 2 and 3.

## 4. Discussion

The results of this study showed that NMES is effective for muscle training in sport (we observed intensive increase of power and mass of quadriceps muscle after one month therapy), which corresponds with some research studies. However, experiments in the literature are usually based on small number of participants, unclear randomization (researchers do not use validated methods of randomization such as a computer or marked envelopes, only physician decides the allocation of participants), lack of inclusion and exclusion criteria, and no assessment of the safety of NMES application in sport [11, 13, 14, 19].

Paillard et al. [11] in their study observed the effects of different types of NMES programs on vertical jump performance. Twenty-seven healthy trained male students in sports-sciences were recruited and randomized into three groups. The control group (C group, *n* = 8) did not perform NMES training. Two other groups underwent 3 training sessions a week for over 5 weeks on the quadriceps femoris muscle—F group (*n* = 9): stimulation with an 80 Hz current for 15 min for improving muscle strength; E group (*n* = 10): stimulation with a 25 Hz current for 60 min for improving muscle endurance. The height of the vertical jump was measured before NMES training (test 1) and one week (test 2) and five weeks (test 3) after the end of the programs. The results showed that the height of the vertical jump significantly increased in both the F and E groups between tests 1 and 2 (5 cm and 3 cm, resp.). Results of test 3 showed that both groups preserved their gains. In authors opinion a NMES training program improves muscle strength.

British researchers [19] observed prolonged NMES in sedentary adults. Fifteen healthy subjects (10 men, 5 women) with a sedentary lifestyle completed a 6-week training program during which they completed an average of 29 1-hour of NMES sessions. The form NMES used by the subjects was capable of eliciting a cardiovascular exercise response without loading the limbs or joints. It achieved this by means of inducing rapid, rhythmical contractions in the large leg muscles. A crossover study design was employed with subjects undergoing their habitual activity levels during the nontraining phase of the study. The training effect was evaluated by means of a treadmill test to determine peak aerobic capacity, peak oxygen consumption, Vo_2_, a 6-minutes walking distance test, and measurement of body mass index (BMI) and quadriceps muscle strength. At baseline, the mean values for peak Vo_2_, 6-min walking distance, quadriceps strength, and BMI were 2.46 l/min, 493.3 m, 360.8 N, and 26.9 kg/m^2^, respectively. After training, subjects demonstrated statistically significant improvements in all variables except BMI. Peak Vo_2_ increased by an average of 0.24 l/min (*P* < 0.05), walking distance increased by 36.6 m (*P* < 0.005), and quadriceps strength increased by 87.5 N (*P* < 0.005). These results suggest that NMES can be used in sedentary adults to improve muscle strength.

Snyder-Mackler et al. [13] included 85 patients to treat with highintensity NMES, high-level volitional exercise, low-intensity NMES, or combined high and lowintensity NMES. All treatment was performed isometrically with the knee in 65 degrees of flexion. All of the patients participated in an intensive program of closed-kinetic-chain exercise. After four weeks of treatment, the strength of the quadriceps femoris muscle and the kinematics of the knee during stance phase were measured. Quadriceps strength averaged 70% or more of the strength on the uninvolved side in the two groups that were treated with high-intensity NMES (either alone or combined with low-intensity NMES), 57% in the group that was treated with high-level volitional exercise, and 5% in the group that was treated with low-intensity NMES. The kinematics of the knee joint were directly and significantly (*P* < 0.05) correlated with the strength of the quadriceps.

Other researchers [14] presented 27 healthy subjects (mean age 23.4 years) volunteered for the study and were randomly assigned to 1 of 3 groups; control group (no NMES); group 2 (NMES 2 times per week); and group 3 (NMES 3 times per week). Groups 2 and 3 received NMES (10 minutes per session) over a 4-week period for a total of 8 and 12 NMES training sessions, respectively. The isometric quadriceps femoris muscle force produced during NMES was monitored during each treatment minute. The force of the quadriceps femoris was assessed prior to the first week and at the start of weeks 2, 3, and 4 of the 4-week training program, with a final measurement after the fourth week (5 total measurements) for all subjects. Only the mean percent change in quadriceps power before and after the 4 weeks of training with NMES between the control group and group 3 was significantly different (*P* = 0.021).

In our research we studied motion and function parameters in knee, without analysis of inflammatory reaction or quadriceps muscle damage.

A review of the literature can be found to have only one article about muscle pathologies after NMES application. Vanderthommen et al. [17] studied the effects on muscle function of an electrical stimulation bout applied unilaterally on thigh muscles in healthy male volunteers. One group (ES group, *n* = 10) received consecutively 100 isometric contractions of quadriceps and 100 isometric contractions of hamstrings (on-off ratio: 6-6 s) induced by neuromuscular electrical stimulations (NMES). Changes in muscle torque, muscle soreness (VAS), muscle stiffness, and serum creatine kinase (CK) activity were assessed before the NMES exercise (preex.) as well as 24 h, 48 h and 120 h after the bout. A second group (control group, *n* = 10) was submitted to the same test battery as the stimulation group and with the same time-frame. The between group comparison indicated a significant increase in VAS scores and in serum levels of CK only in the ES group. In the ES group, changes were more pronounced in hamstrings than in quadriceps and peaked at 48 h (quadriceps VAS scores = 2.20 ± 1 (0 at preex.); hamstrings VAS scores = 3.15 ± 2.14 (0 at preex); hip flexion angle = 62 ± 5° (75 ± 6° at preex.); CK activity = 3021 ± 2693 IU · l − 1 (136 ± 50 IU · l − 1 at preex.). The results of the study suggested the occurrence of muscle damage that could have been induced by the physiologically incorrect muscle recruitment in NMES and the resulting overrated mechanical stress.

In our study the NMES appeared to be safe for biomechanics of knee joint. Based on our previous experience we can strongly recommend the goniometry pendulum test as useful diagnostic method in physical therapy process [20]. The purpose of this clinical study was to assess low frequency, low intensity magnetic fields in the enhancement of the physical rehabilitation of patients after knee endoprosthesis surgery. The study included 62 patients who underwent total knee arthroplasty. Group A consisted of 32 patients who were physically rehabilitated. Group B consisted of 30 patients who were physically rehabilitated and treated additionally with pulsing magnetic fields (5 mT, 30 Hz, 20 min once a day, 5 days weekly). Therapy lasted for 3 weeks for both groups. The rehabilitation process was evaluated using a goniometer, tensometer, goniometry pendulum test, Lysholm scale for knee function, and a visual analogue scale (VAS) questionnaire for pain and activity. The pendulum test appeared very useful for precise observation of biomechanical changes in knee joint during therapy process. The physical therapy decreased the logarithm decrement of suppression (*λ*) by about 7.9% in group A and 9.6 in group B, suppression index (*β*) decreased; about 11% in group A and 13.3% in group B. The period of oscillations increased by 4.9% in group A and 4% in group B; number of oscillations (*n*) increased by 22.1% in group A and 20.6 in group B. The whole time of oscillations increased of 29.8% in group A and 24.5% in group B.

In our study we did not observe both early and long term results (goniometry pendulum testour research is the first attempt in literature of using this apparatus in sport) any pathological changes in knee function after increasing the strength and mass of quadriceps in use of NMES. The progress in muscle parameters did not have influence pathologically on knee function.

### 4.1. Limitations of Study

In our study soccer players after a month of therapy with NMES + rehabilitation or only rehabilitation program (7 months after ACL reconstruction) returned to sport and league competition, what could interfere with follow-up observation. We were not able to demonstrate a relationship between the pendulum test and muscle dysfunction either in the study or from previous research using a similar protocol correlated with markers of muscle damage. We tried to use objective measurement methods, but we did not collect data related to functional outcomes, of which there are some they could have chosen (triple-hop, crossover-hop, and jump-landing test).

## 5. Conclusion

The results of this study show that NMES (presented parameters in experiment) is useful for strengthening the quadriceps muscle in soccer athletes. There is an evidence the benefit of the NMES in restoring quadriceps muscle mass and strength of soccer players. In our study the neuromuscular electrical stimulation appeared to be safe for biomechanics of knee joint. The pathological changes in knee function were not observed.

## Figures and Tables

**Table 1:** Characteristics of individuals from group A and B.

	Group A	Group B	Level of significance
Number of participants	40	40	**P > 0.05
Age (years)			
Range	17–29	17–29	*P > 0.05
Mean	22.4	21.3	
SD	5.78	5.67	
Operated knee			
Right	26	23	**P > 0.05
Left	14	17	
Height (cm)			
Range	171–193	170–186	*P > 0.05
Mean	180.3	176.5	
SD	7.54	8.11	
Weight (kg)			
Range	71–80	63–100	*P > 0.05
Mean	73.8	70.9	
SD	5.76	7.12	
BMI (kg/m^2^)			
Range	19.2–25.8	20.3–28.9	*P > 0.05
Mean	22.4	23.4	
SD	4.22	3.03	
Muscle strength (N)			
Operated	578.4–701.3	567.5–689.9	**P > 0.05
Range	645.9	648.6	
Mean	34.6	38.6	
SD			
		
Nonoperated			
Range	821.4–879.8	831.5–889.7	
Mean	840.1	840.4	
SD	23.4	27.9	
Duration of career (how long soccer players practice their discipline) in years			
Range	3.4–10.5	3.3–8.5	*P > 0.05
Mean	5.4	4.8	
SD	2.46	1.89	

*the *t*-test, **the *χ* ^2^-test.

**Table 2:** Goniometry pendulum test in group A.

Parameter	Before therapy		After therapy		Followup				Level of significance
					1 month		3 months		
	Mean	SD	Mean	SD	Mean	SD	Mean	SD	
*λ*									
Operated	0.57	0.19	0.56	0.16	0.55	0.20	0.55	0.22	*P* > 0.05
Nonoperated	0.47	0.18	0.45	0.19	0.46	0.22	0.46	0.22	*(in all comparisons)
*β* [s^−1^]									
Operated	0.77	0.30	0.77	0.28	0.74	0.22	0.75	0.30	*P* > 0.05
Non-operated	0.57	0.30	0.56	0.31	0.53	0.23	0.54	0.30	*(in all comparisons)
*T* [s]									
Operated	0.89	0.30	0.94	0.30	0.95	0.27	0.93	0.30	*P* > 0.05
Non-operated	0.95	0.22	0.99	0.19	0.99	0.19	0.98	0.21	*(in all comparisons)
*n*									
Operated	3.32	1.22	3.32	1.43	3.31	1.21	3.32	1.25	*P* > 0.05
Non-operated	4.78	1.22	4.87	1.45	4.86	1.21	4.87	1.21	*(in all comparisons)
*t* [s]									
Operated	2.07	1.22	2.07	1.52	2.12	1.10	2.13	1.21	*P* > 0.05
Non-operated	3.04	1.20	3.04	1.45	3.01	1.13	3.01	1.13	*(in all comparisons)

*Analysis of variance ANOVA.

Legends:

*λ*: logarithm decrement of suppression (more than 0.65 is pathology of I Outebridge degree of the articular cartilage), *β*: suppression index (more than 0.85 is pathology of I Outebridge degree of the articular cartilage), *T*: period of oscillations (less than 0.75 is pathology of I Outebridge degree of the articular cartilage), *n*: number of oscillations (less than 3.00 is pathology of I Outebridge degree of the articular cartilage), and *t*: whole time of oscillations (less than 1.50 is pathology of I Outebridge degree of the articular cartilage).

**Table 3:** Goniometry pendulum test in group B.

Parameter	Before therapy		After therapy		Followup				Level of significance
					1 month		3 months		
	Mean	SD	Mean	SD	Mean	SD	Mean	SD	
*λ*									
Operated	0.55	0.21	0.58	0.19	0.56	0.22	0.55	0.20	*P* > 0.05
Nonoperated	0.48	0.21	0.43	0.19	0.44	0.22	0.45	0.20	*(in all comparisons)
*β* [s^−1^]									
Operated	0.75	0.32	0.76	0.21	0.75	0.22	0.75	0.28	*P* > 0.05
Non-operated	0.58	0.27	0.57	0.28	0.55	0.25	0.54	0.28	*(in all comparisons)
*T* [s]									
Operated	0.87	0.34	0.90	0.35	0.90	0.29	0.90	0.30	*P* > 0.05
Non-operated	0.95	0.19	0.97	0.19	0.97	0.19	0.99	0.20	*(in all comparisons)
*n*									
Operated	3.35	1.31	3.33	1.38	3.33	1.30	3.33	1.30	*P* > 0.05
Non-operated	4.75	1.31	4.79	1.39	4.80	1.41	4.80	1.41	*(in all comparisons)
*t* [s]									
Operated	2.11	1.03	2.09	1.04	2.10	1.11	2.11	1.01	*P* > 0.05
Non-operated	3.10	1.28	3.11	1.25	3.11	1.11	3.10	1.09	*(in all comparisons)

*Analysis of variance ANOVA.

Legends:

*λ*: logarithm decrement of suppression (more than 0.65 is pathology of I Outebridge degree of the articular cartilage), *β*: suppression index (more than 0.85 is pathology of I Outebridge degree of the articular cartilage), *T*: period of oscillations (less than 0.75 is pathology of I Outebridge degree of the articular cartilage), *n*: number of oscillations (less than 3.00 is pathology of I Outebridge degree of the articular cartilage), and *t*: whole time of oscillations (less than 1.50 is pathology of I Outebridge degree of the articular cartilage).
